# Identifying outbreaks of Porcine Epidemic Diarrhea virus through animal movements and spatial neighborhoods

**DOI:** 10.1038/s41598-018-36934-8

**Published:** 2019-01-24

**Authors:** Gustavo Machado, Carles Vilalta, Mariana Recamonde-Mendoza, Cesar Corzo, Montserrat Torremorell, Andrez Perez, Kimberly VanderWaal

**Affiliations:** 1 Department of Population Health and Pathobiology, College of Veterinary Medicine, Raleigh, North Carolina USA; 2 Department of Veterinary Population Medicine, University of Minnesota, St. Paul, Minnesota USA; 3 Institute of Informatics, Universidade Federal do Rio Grande do Sul, Porto Alegre, Rio Grande do Sul, Brazil

## Abstract

The spread of pathogens in swine populations is in part determined by movements of animals between farms. However, understanding additional characteristics that predict disease outbreaks and uncovering landscape factors related to between-farm spread are crucial steps toward risk mitigation. This study integrates animal movements with environmental risk factors to identify the occurrence of porcine epidemic diarrhea virus (PEDV) outbreaks. Using weekly farm-level incidence data from 332 sow farms, we applied machine-learning algorithms to quantify associations between risk factors and PEDV outbreaks with the ultimate goal of training predictive models and to identify the most important factors associated with PEDV occurrence. Our best algorithm was able to correctly predict whether an outbreak occurred during one-week periods with >80% accuracy. The most important predictors included pig movements into neighboring farms. Other important neighborhood attributes included hog density, environmental and weather factors such as vegetation, wind speed, temperature, and precipitation, and topographical features such as slope. Our neighborhood-based approach allowed us to simultaneously capture disease risks associated with long-distance animal movement as well as local spatial dynamics. The model presented here forms the foundation for near real-time disease mapping and will advance disease surveillance and control for endemic swine pathogens in the United States.

## Introduction

The circulation of endemic and epidemic viruses in livestock populations at farm, regional, and national levels has significant impact on animal welfare and livestock production within the United States and globally^1^. Numerous mechanisms play an apparent role in the spread of viruses between farms, including the movement of infectious animals, airborne spread of aerosols, wildlife, and contaminated fomites (i.e., trucks, equipment, boots), feed and personnel^2–5^. However, the relative importance of those drivers for between-farm transmission remains poorly understood. Movement of animals into a farm, for example, clearly increases the probability of disease introduction^6^, and understanding the network of livestock movements and analyzing the routes, volumes, frequency, and risks associated with animal movement is a prerequisite effective preventive, control, and disease surveillance measures in livestock^6–8^. At the same time, local spread of viruses between neighboring farms in close spatial proximity has been repeatedly demonstrated, perhaps mediated by windborne or fomite-related mechanisms^9,10^. A farm’s risk of infection may thus be impacted not just by its own animal movements, but also by movements made by neighbors. These local spatial dynamics are rarely accounted for in network-based assessments of risks associated with livestock movement, although such dynamics are an emergent property of epidemiological models simulating disease spread in spatial networks^11,12^.

Much of our understanding of between-farm pathogen transmission in the United States swine industry is based on outbreak investigations, case-control studies, cohorts and case reports involving a relatively small number of farms^13–15^. Large-scale datasets in which to investigate the interacting roles of animal movements versus local transmission in between-farm spread are largely lacking. This is especially true for endemic diseases, where farm-level occurrences are often not tracked, and for countries in which no regional or national-level databases are available for livestock premise locations and animal movements. In the United States, limited data exists on the structure and connectivity of livestock movement networks because reporting the movement of animals between farms is not required as long as movements do not cross state boundaries. Swine production companies, or “systems,” are vertically integrated, with pigs generally moving from sow farms to nurseries to finishing farms. However, movements are not tracked by governmental agencies responsible for animal health. In contrast to many other developed and middle-income countries, the structure and connectivity of the swine industry has only been described to a limited extent^16,17^.

In this study, we explored how the occurrences of porcine epidemic diarrhea virus (PEDV) in sow farms is influenced by the potential interacting effects of animal movement (movements into neighboring farms), local spatial spread, and environmental conditions. Briefly, PEDV is a RNA coronavirus in the family *Alphacoronaviridae* that causes high pre-weaning mortality in piglets^18^. The virus emerged in the United States in 2013 and rapidly spread to 30 states infecting approximately 50% of breeding herds by the middle of 2014^19^. PEDV transmission among individual pigs occurs primarily by direct and indirect contact via the fecal-oral route^19,20^. Recent research has examined the role of animal transport in the spread of PEDV^21–23^. An analysis of the animal movement network for one swine system showed the frequency of movements declined during the initial emergence of PEDV^24^. Indirect transmission between farms may also be possible via airborne mechanisms^25,26^. Potential environmental conditions that may facilitate virus survivability in the environment include temperature^27^ and relative humidity^28^, but these environmental factors have not been integrated into broader analyses of risk factors for transmission. More generally, there is a lack of quantitative knowledge about the impact of meteorological, environmental, and topographic features (i.e. land cover, slope) on PEDV spread, though recent work on porcine reproductive and respiratory syndrome virus (PRRSV) suggests that there are potential relationships between environmental and topographic variables and between-farm virus transmission^29^.

To enhance our understanding of the interacting variables that contribute to the circulation of PEDV between farms, we analyzed a large retrospective dataset containing weekly incidence of PEDV in sow farms over the course of 12 months in relation to pig movements and meteorological, environmental, and topographic variables. For this study, we define a neighborhood as a 10 km radius around each sow farm. First, we describe flows of pig movements into and between neighborhoods. Our second and main objective was to quantify the weekly risk of PEDV outbreaks in sow farms based on animal movements and the likelihood of exposure from neighboring farms. We evaluate the relative importance of “neighborhood effects” in determining infection risk, including animal movements into nearby farms, local hog density, environmental factors, and landscape structure. We used machine learning algorithms to model how potential predictors impacted whether a sow farm became infected, referred to as an outbreak, in each one-week period over the course of one year. We also identified the most influential variables on disease risk and evaluated the predictive accuracy of our models for predicting new outbreaks. Our approach for analyzing neighborhood effects on between-farm transmission significantly advances the understanding of PEDV epidemiology and spread and provides a powerful framework for analyzing the joint effect of patterns of animal movement and environmental factors on infection risk in livestock populations.

## Results

### Descriptive analysis

**Figure 1:**
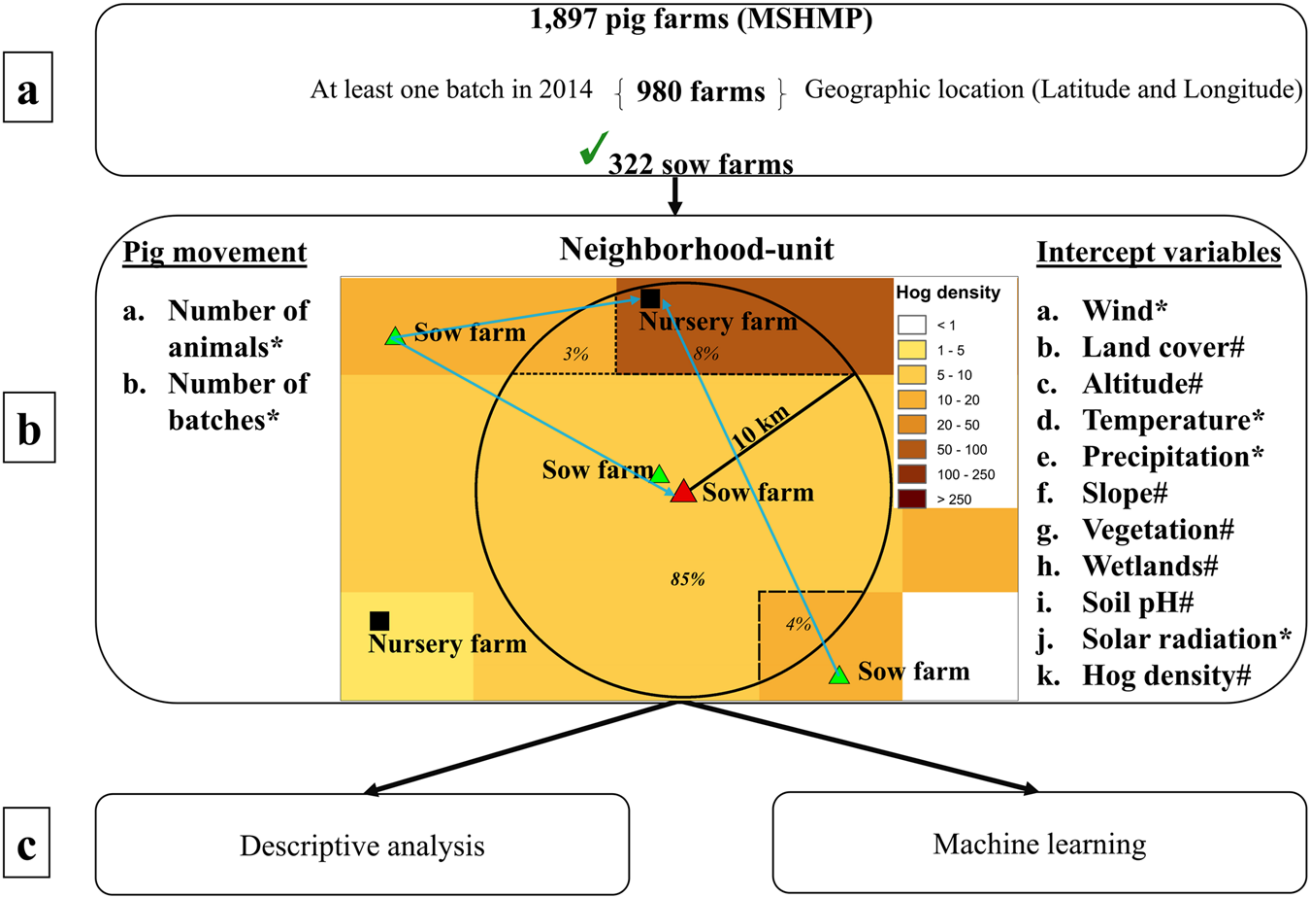
Study pipeline: (**a**) From the total of 1,897 production farms in the original database, 980 farms matched the inclusion criteria of having received at least one shipment of live pigs during 2014 and from which spatial coordinates were available. (**b**) A neighborhood was defined as the 10 km radius around each sow farm. Incoming pig movements are represented by blue lines, and were summed for all farms receiving pigs within a given neighborhood. Environmental/landscape variables (e.g. hog density): if the data layer have categorical nature, the variable was defined as the majority class (by area) within the neighborhood; if the data layer was a continuous raster, the variable was defined as the mean value within the neighborhood. (**c**) Descriptive analysis and machine learning approaches were used to explore the influence of neighborhood effects on outbreaks within the focal sow farm. *Dynamic variables (monthly means were calculated); ^#^Static variables (for categorical variables, the major class was identified; if numeric, the mean was calculated-see Supplementary Information S2 for more details); for movements, the number of animals and number of batches were calculated each week.

A retrospective longitudinal study was conducted using data from three spatially overlapping swine production systems in the United States, representing a large majority of commercial farms in the area. Daily live pig movements were available from two out of the three systems. Data from 1,897 farms, including all production types (sow farms, nursery farms, finishing, and others) were available from the Morrison Swine Health Monitoring Project (MSHMP) database (Fig. 1). Approximately half (n = 980, 51.7%) of those farms met the inclusion criteria, namely, a) availability of spatial coordinates (latitude and longitude), and b) record of at least one shipment of pigs into the farm during 2014 (i.e., premises with no movements were assumed to be inactive farms). In addition, we excluded sow farms (n = 173) located outside the study area. Of the remaining farms included in the study, 332 were sow farms (i.e., consequently 332 neighborhoods).

The study period was defined as the period of time in which animal movement data were available for two of the three systems and included a full year (2014). Movement data included: (a) farm of origin; (b) farm of destination; (c) shipment date; (d) number of pigs moved; and (e) type of pigs moved (movement classification). Movement classifications included: (1) breeding movements: all movements from gilt development units (GDU) to sow farms to replace breeding stock; (2) weaning movements: movements of weaned piglets from sow farms to nurseries or wean-to-finish farms; (3) finisher movements: movements of growing pigs from nurseries to finishing farms, and (4) internal movements: transition of pigs from one building/barn/ to another within the same site that may involve pigs being briefly outside of enclosed spaces. Movements of pigs to slaughterhouses or cull sow stations were not included, as these were considered to be an epidemiological dead-end; there were also no slaughterhouses or cull sow stations located within any neighborhood.

**Figure 2:**
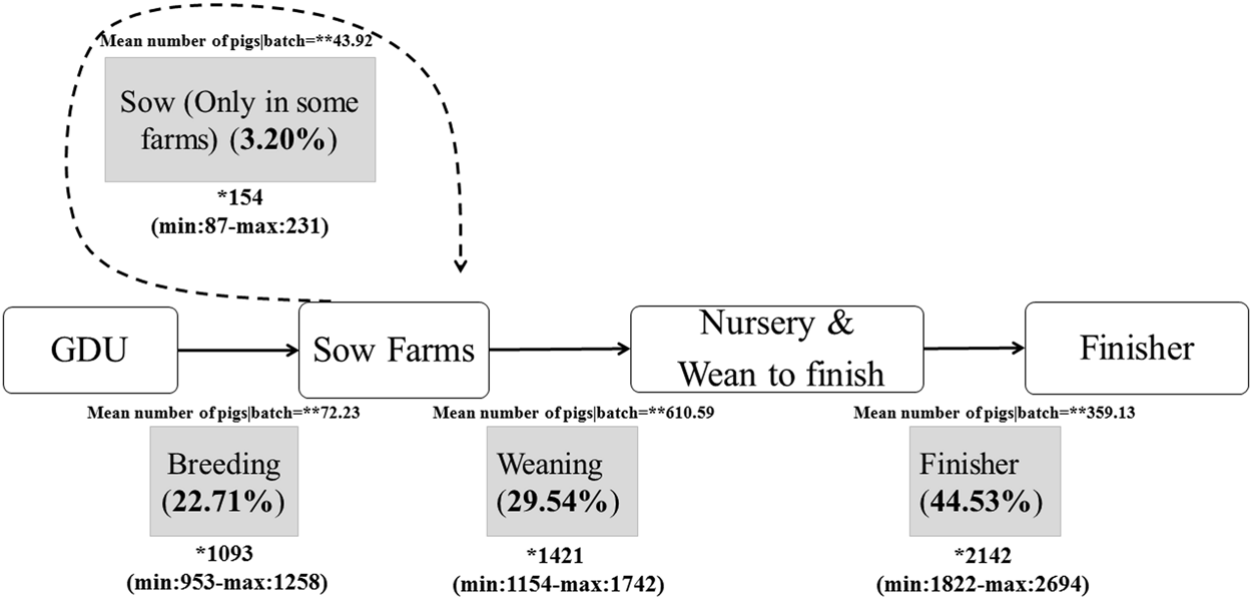
Simplified representation of pig movements in swine production systems. Overall percentage of the number of shipments associated with each type of movement are represented in parentheses. *Represents the monthly mean (min-max) number of batches for each movement type, and **represent the overall mean number of pigs shipped per batch.

A quantitative summary of swine movement data from this study is shown in Fig. 2. More than 15 million pigs in 44,238 batches were transported in 2014. As expected many of movements (44.5%) were from nurseries to finishers or wean-to-finish farms, with a monthly average of 2,142 batches and mean of 359 pigs/batch. Movements of weaned pigs accounted for 29.5% of all movements with a monthly average of 1,421 batches and approximately 610 pigs/batch. Breeding movements were less predominant, accounting for 22.7% of movements with an average of 1,093 batches monthly. Movements within sow farms (internal movements) accounted for only 3.2% and 154 batches, with a mean of 44 animals.

**Figure 3:**
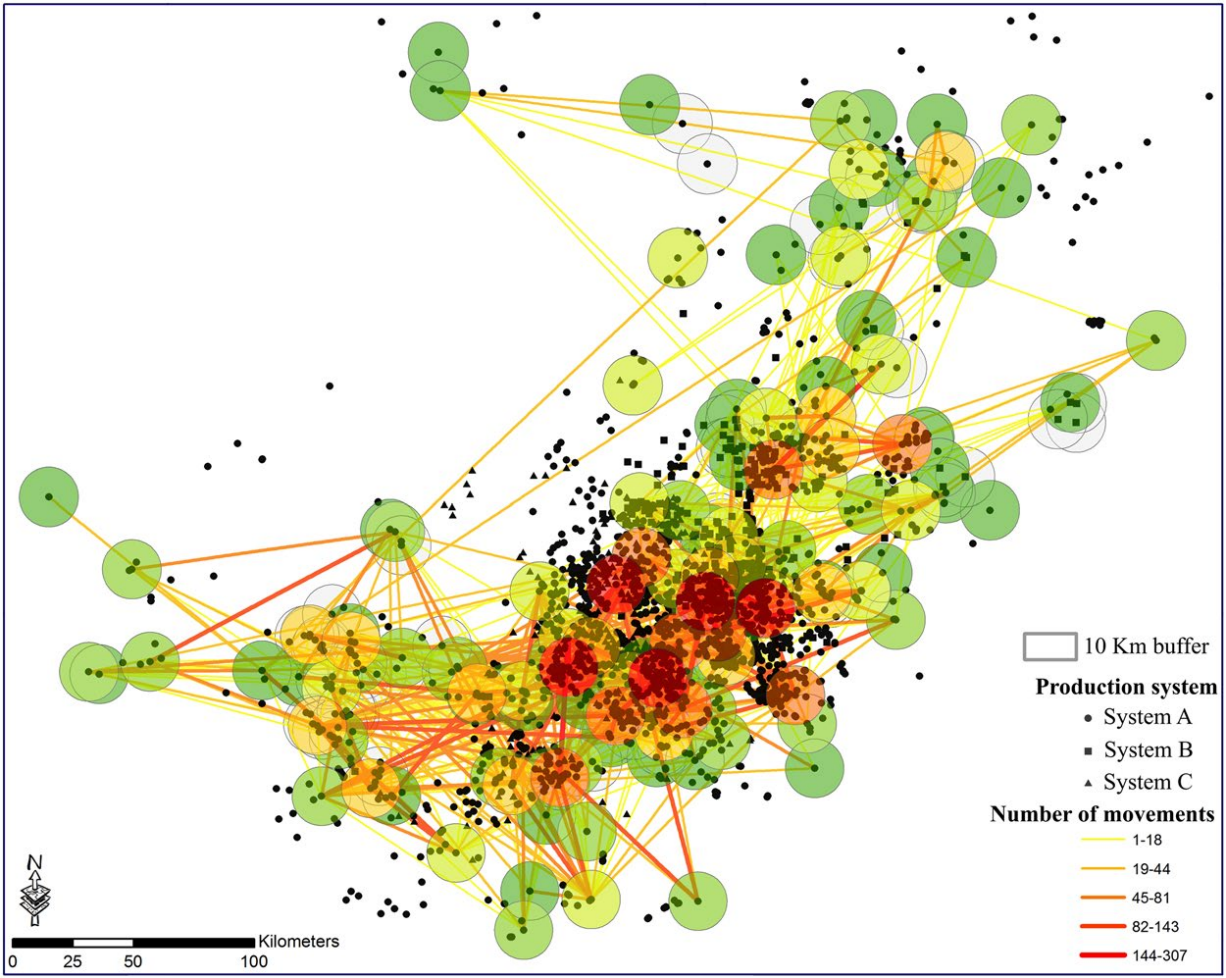
Distribution of farms in the study area. Each black symbol represents a swine farm and each circle represents a neighborhood around a sow farm and redder colors indicate more movements into that neighborhood. The aggregated number of batches between neighborhoods for the first week of 2014 are represented as lines connecting neighborhoods, where color corresponds to the number of batches moved.

The second objective of this study was to analyze the likelihood that a sow farm was infected with PEDV each week during the course of 52 weeks given the local environmental, landscape, and behavior of nearby farms (i.e., animal movements). For each week, farms were classified as “0” if they were at-risk and thus susceptible to infection, and “1” if they reported clinical PEDV. Our goal was to predict when a farm transitioned from 0 to 1. Once a farm was infected, it was removed from that database as it was no longer considered at-risk. Given the one year duration of the study, farms were not allowed to return to 0. A 10 km radius around a sow farm was defined as a “neighborhood” based on reported distances of potential airborne spread of swine viruses such as PEDV and PRRS^25,26,30–32^. A spatial representation of sow farm neighborhoods and flows of pig movements between these neighborhoods in the first week are plotted in Fig. 3. It is important to note that network visualizations provide a simplified view of the true dynamics of the network, and details of the day-to-day changes in the network and directionality of links are not shown. Further, we represent movements as a straight line (Euclidean distance) between neighborhoods rather than the actual transit distance of trucks on roads. For each sow farm, the 2014 PEDV outbreak history and all animal movements into neighboring farms as well as environmental and landscape variables at the level of the neighborhood was obtained (Fig. 3, see Supplementary Material).

**Figure 4:**
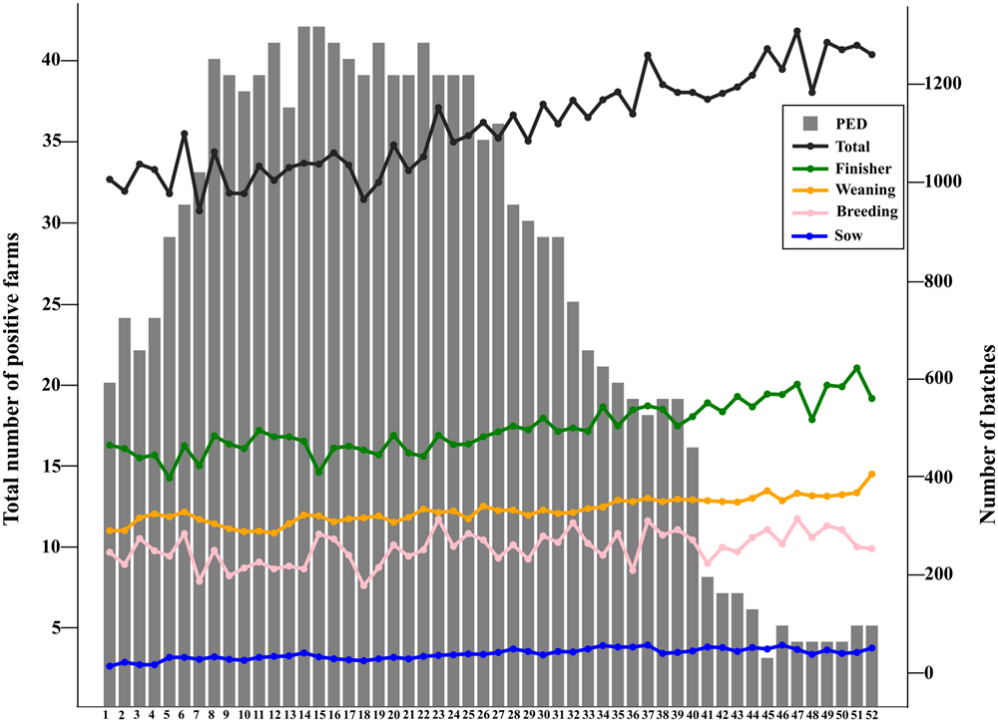
Total number of Porcine Epidemic Diarrhea virus-positive farms and total number of movements into neighborhoods per week in 2014. Number of movements by movement type are represented as colored lines.

In total (n = 35, 10.5%) sow farms experienced outbreaks at some point during 2014. The number of pigs shipped into neighborhoods each week, along with total number of PEDV positive farms over time, are shown in Fig. 4. The number of PEDV-positive farms reach the maximum during week 14 and 15 (April 2014), followed by gradual decrease. By October, less than ten neighborhoods were experiencing PEDV outbreaks. Although the number of batches per week increased slightly towards the end of 2014, movement frequencies did not seem to be substantially affected by the epidemic.

### Model validation and selection of best machine learning algorithm

Machine learning models were trained to classify (yes/no) whether a sow farm would experience a PEDV outbreak in a given week given a set of covariates. Briefly, models were trained using 80% of the data; the remaining 20% of observations were withheld from model building in order to provide a quasi-independent data set to validate the accuracy of model predictions. Ten-fold cross-validation using the training data was used to check the model’s accuracy. Machine learning algorithms applied included random forest (RF), support vector machines (SVM), and gradient boosting machines (GBM).

Variables that were hypothesized to influence the occurrence of PEDV outbreaks in sow farms were the total number of live pigs moved between neighborhoods, the number of batches of pigs moved between neighborhoods according to movement classification (finisher, weaning, breeding, or internal), hog density within the neighborhood, and 11 geo-environmental variables such as vegetation, land cover, wetlands area, global relief (altitude), global topography (slope), average solar radiation, average precipitation and temperature, topsoil pH, major soil group classification, and mean wind speed, and season of the year (fall: Oct-Dec, winter: Jan-Mar, spring: Apr-Jun, summer: Jul-Sep). A full list of variables, data sources, and their temporal nature is provided in the Supplementary Information S1 and S3. These variables were all summarized at the neighborhood level and used as covariates in machine learning algorithms (see Supplementary Information S2 for more detail).

**Figure 5:**
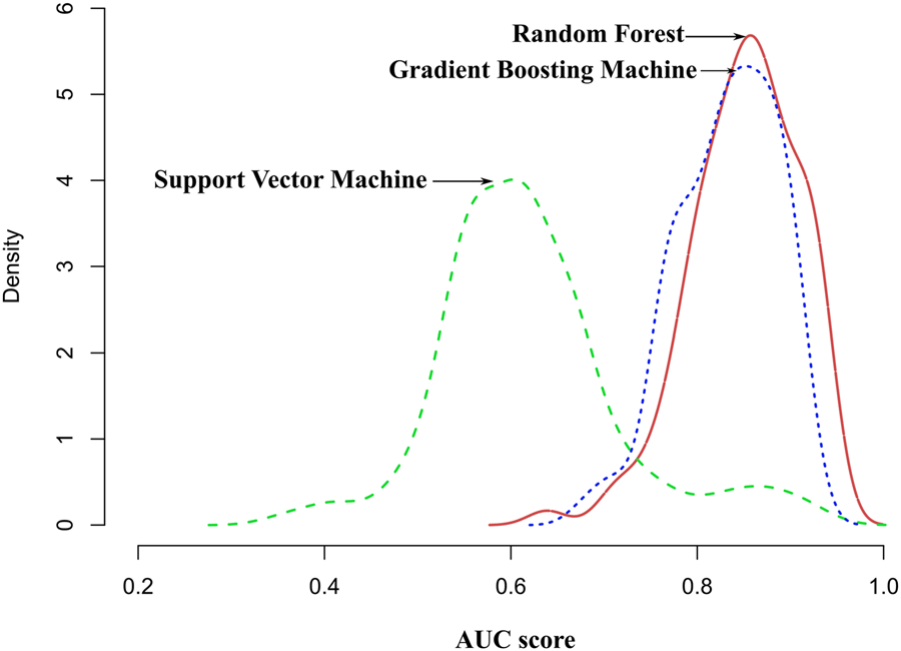
Comparative evaluation of algorithms based on repeated cross-validation. The performance of the model averaged over 10-fold-cross-validation are summarized by a kernel density estimator.

The best-performing algorithm was selected by comparing model performance in the cross-validation step and ranking algorithms according to area-under-the-curve (AUC) scores, accuracy, sensitivity, and specificity (Fig. 5). Random Forest (RF) showed the highest performance, with a narrow and slightly right-shifted AUC distribution compared to Gradient Boosting Machine (GBM, Fig. 5). Support Vector Machine (SVM) had lower AUC values with a much wider distribution, indicating poorer and more unstable performance in contrast to other methods. The confusion matrix for all algorithms trained with the complete set of variables, averaged over the 10 repetitions of the 10-fold cross-validation, was used to generated accuracy, sensitivity and specificity. We obtained a model accuracy of 83.0% (±2.3) for RF, 80.0% (±3.2) for SVM, and 79.0% (±11.4) for GBM. Sensitivities were 83.3% (±2.3) for RF, 80.5% (±15.5) for SVM, and 78.6% (±2.6) for GBM. Specificities were 79.6% (±2.5) for RF, 78.2% (±8.3) for SVM, and 80.0% (±4.3) for GBM.

In addition to evaluating the algorithms performance via the cross-validation resampling procedure based on training data, we also assessed their predictive performance for the quasi-independent test data (these results are from a single data partitioning of the original dataset, corresponding to 20% of observations). The AUC scores obtained for the algorithms were ~96% for RF and GBM and 92% for SVM. Accuracy estimates for the holdout test data were 97.0% (CI_95%_: 96.0–98.1%) for RF, 92.4% (CI_95%_: 91.0–93.8%) for SVM, and 93.0% (CI_95%_: 92.2–97.8%) for GBM. Finally, RF reached the most balanced sensitivity and specificity in this test, with 65.2% and 95.0%, respectively. SVM had a sensitivity of 35.5% and specificity of 90.0%, whereas GBM achieved sensitivity of 51.0% and specificity of 97.0%.

### Variable importance

**Figure 6:**
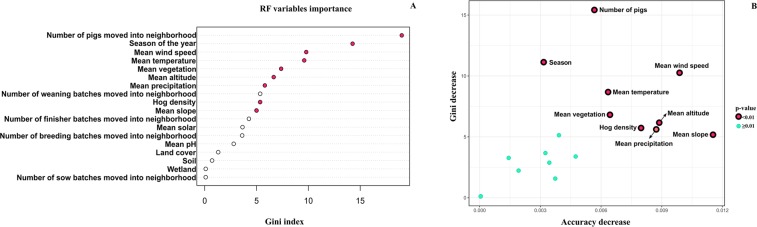
Variable importance plot. (**A**) Variables were ordered according to Gini index unscaled values. Red dots represented the variables that were used for splitting the data more often than it would happen by chance. (**B**) Showing variable importance according to the Gini index and the accuracy decrease metric. Variables in red represent ones that were used for splits in the random forest more often that random. *Sow, finisher, weaning, breeding correspond to number of batches moved with this classification (see Fig. 2 for a description of the volume of movements).

Random forest was the best algorithm therefore used for the all further analysis. For the variable importance analysis, the eighteen variables used for model training were ranked according to their contribution to model predictions, as measured by the Gini index which represents the unscaled average decrease in node impurity (Fig. 6A). The variable importance score (Gini Index) represents how relevant each variable was for predicting PEDV outbreaks, with larger values representing more important predictors. We also applied several additional approaches to select the most critical variables for the prediction algorithm. We calculated the “accuracy decrease” as the extent to which the accuracy of predictions changed when an explanatory variable was permuted relative to the outcome (Fig. 6B). In addition, we calculated a p-value for each variable that were hypothesized to influence the occurrence of PEDV outbreaks (Fig. 6B) to evaluate whether it was used in the random forest more often than if variables were selected at random (see methods^33,34^). The combination of Gini Index and accuracy decrease (see Fig. 6, y-axis: Gini decrease and x-axis: accuracy decrease) shows a natural division in importance amongst variables, with nine variables (highlighted in red in Fig. 6) revealed to be the most critical. The effect of these variables on the outcome was further explored via partial dependence plots, which measured the marginal effect of each variable on the outcome after controlling for the effects of all others.

**Figure 7:**
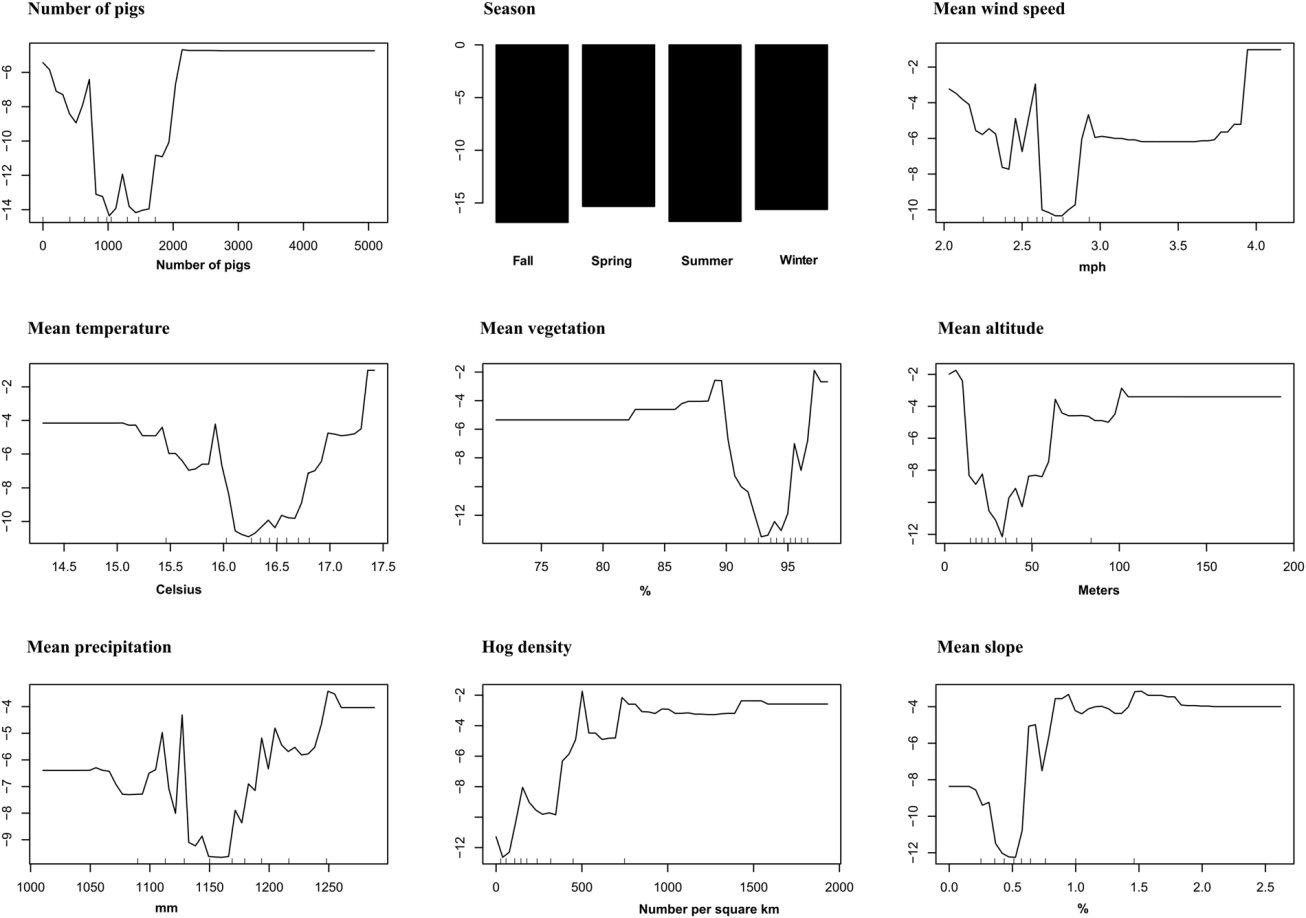
Partial dependence plots of the marginal effect of the top nine variables on the probability of a PEDV outbreak. The y-axis represents a log scale (the logit function gives the log-odds, or the logarithm of the odds p/(1-p)).

**Figure 8:**
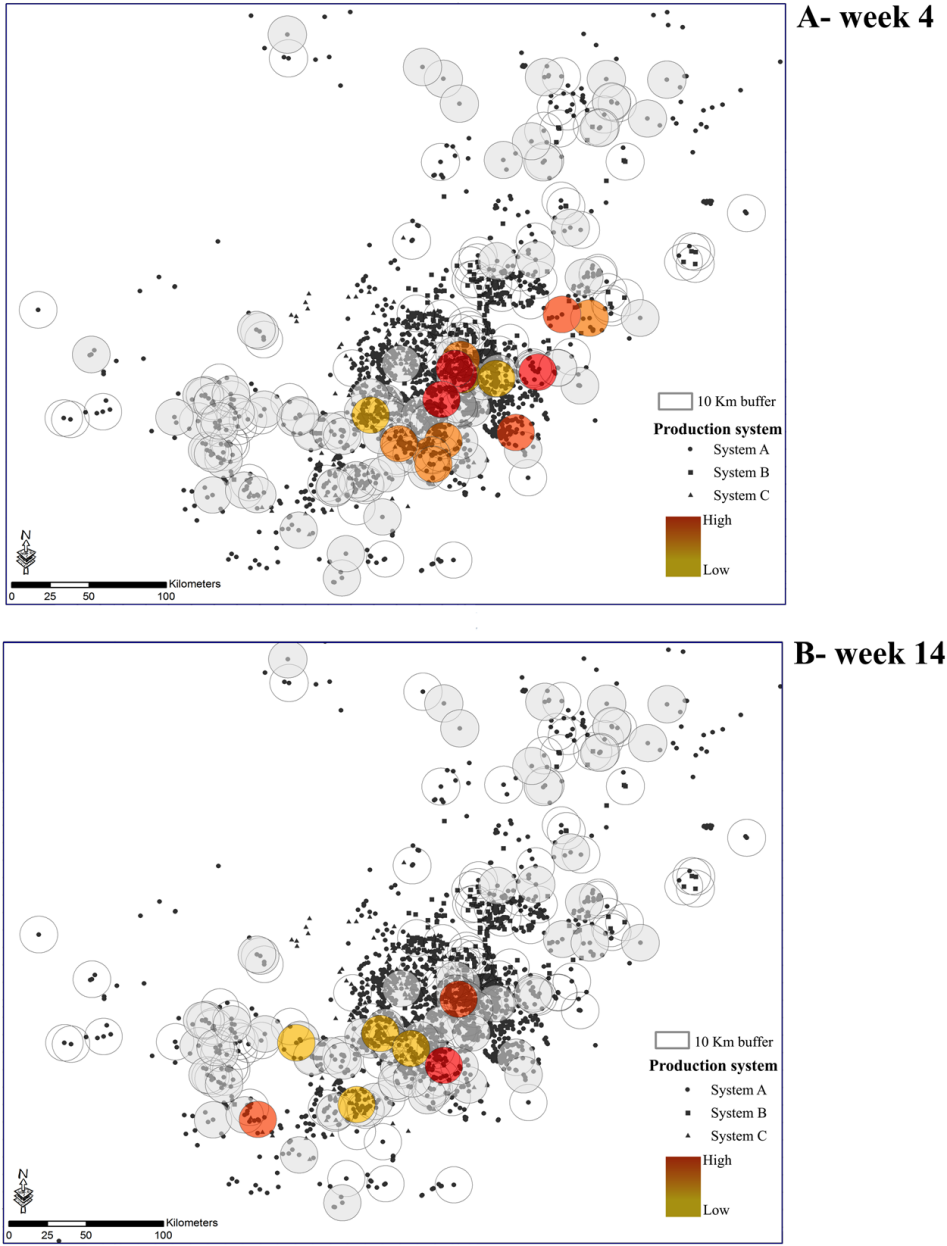
Risk maps generated through the selected algorithm- Random Forest. The black symbol represents a swine farm, each circle represents a neighborhood, and hotter colors represent predictions of a PEDV outbreak for a given week. (**A**) week 4 for 2014 season and (**B**) for week 14 of 2014 season.

The most important predictor for PEDV outbreaks was the overall number of pigs moved into the neighborhood, followed by season of the year and other climatic and environmental covariates. The most critical variables are represented in partial dependence plots (Fig. 7), which show the marginal effect of each variable on outbreak probability after controlling for the effects of all other predictors. Higher values on the y-axis indicated higher probabilities of PEDV outbreaks. In some partial dependence plots, the relationship between x and y is relatively flat, which is probably due to small sample size for some ranges of the x variable. For instance, only a few neighborhood units ~3% had values below 80% for vegetation coverage. We illustrate the spatiotemporal predictions emerging from our model for select weeks: week 4-when the number of observed cases was increasing (Fig. 8a), and week 14-when the PEDV reached its maximum prevalence (Fig. 8b).

## Discussion

A number of studies have examined swine movement networks as a main transmission route for disease spread^16,35–39^, network analysis and epidemiological data have recently been used to assess risk of local areas being exposed to pathogens as a consequence of animal introduction^40^. The approach presented here provides a realistic understanding of PEDV dynamics by capturing long distance spread via animal movements and linking it with epidemiological factors related to local area spread between neighboring farms. We used machine-learning algorithms to uncover factors most likely to predict PEDV outbreaks, providing crucial information toward more effective preventive actions for minimizing the risk of swine infectious diseases. Key predictors of PEDV included the dynamics of swine movements into a sow farm’s neighborhood as well as environmental conditions, such as wind speed, temperature and precipitation (Fig. 6A). The predictions generated by our model constitute an important baseline for disease surveillance and highlight directions for testable hypotheses about environmental influences on disease spread in the swine industry. If implemented, our results suggest that it could be possible to predict PEDV with substantial accuracy, explaining variation in risk even in areas with high farm density (Fig. 8).

As expected for vertically integrated production systems, 75% of between farm movements were associated with transitions between different production stages that occur at different farms (gestation/farrowing in sow farms → nursery or wean-to-finish farms for weaned piglets → finishing farms). In contrast to Lee *et al*.^16^, we did not identify a significant reduction in weekly movements as the PEDV epidemic grew in 2014 (Fig. 4). That result may indicate that the industry may not consider the restriction or alteration of animal movements as a cost-effective or adequate measure to reduce disease spread, and highlights that different companies or geographic regions may have responded differently to the epidemic.

The most important predictor of PEDV was the number of pigs moved into a sow farm’s neighborhood, however, this ranked order as well as the sensitivity of the model could still be different and or improved, maybe by adding other variables that could not be considered here. The number of weaning pig shipments into a neighborhood was moderately ranked when considering the Gini index alone. The importance of weaned piglets originating from sow farms may be related to the dynamics and longer persistence of the virus in sow farms, and perhaps prolonged and heightened infectiousness in young pigs^41^. Future exploration of the importance of the movement of weaned piglets into neighboring farms should be explored as it provides a potentially simpler and streamlined data acquisition process.

The effect of animal movements on risk reported here is consistent with general principles of disease spread in networks^42–46^; livestock movements and network connectivity have been shown to be important for PEDV infection elsewhere^16,37^. In contrast to these studies, the uniqueness of our approach is that we moved beyond simply accounting for the focal farm’s position in the movement network, and instead quantified the influence of movements into spatial neighbors. The hypothesis that movement of animals into a neighborhood influences disease risk in locations other than the farm that received the shipment is consistent with anecdotes by field veterinarians that viral outbreaks in a farm often occur soon after pigs were moved into a neighboring farm. While many movements do not originate from PEDV positive farms, the chance of infectious pigs being transported would increase probabilistically with the number of pigs moved. Our neighborhood-based approach allowed us to capture those local spatial dynamics, which are nearly always overlooked in analyses of livestock movement data. The importance of pigs moving into a neighborhood in our model can be interpreted as increased likelihood of a local introduction of the virus to neighboring farms.

Many environmental features were also important predictors of PEDV outbreaks, suggesting that the likelihood of between-farm spread between neighboring farms is determined in part by aspects of the landscape and weather. Season showed some association with outbreaks, with slightly higher probabilities observed during the winter and spring. However, it is difficult to determine the repeatability of seasonal patterns with only one year of data. Furthermore, seasonal effects did not seem as dramatic as other factors related to spread (Fig. 7).

Another important variable that distinguished outbreaks was the average wind speed. Spatiotemporal patterns of PEDV occurrence have been shown to be consistent with potential airborne spread^26,45^, and PEDV viral RNA can also be recovered from air samples collected up to 16 km from infected farms^25^. Thus, the importance of wind speed is well aligned to other evidence regarding the possibility of airborne-mediated dispersal of the virus between farms, and other weather attributes such as precipitation and temperature may further influence the likelihood of this transmission mode (Fig. 7). However, our results are correlational and it is possible that weather related effects may also influence other potential modes of PEDV introduction to a farm, such as fomites, feed, and trucks^28,36,46–48^.

Our model also found a positive partial dependence between outbreaks and higher hog densities, which is an association that has also been described elsewhere for swine diseases in the United States^49^. Density dependence in transmission dynamics would be expected for most transmissible diseases^50^. However, it is interesting to note that even in areas with a high density of farms, our model still captured fine-scale spatial variation in which specific neighborhoods were at higher risk (Fig. 8). In addition, we identified that neighborhoods with higher altitudes and greater slope were more likely to outbreak with PEDV. This is in contrast to a study that investigated altitude and slope in the epidemiology of PRRSV, where farms in areas with higher slope (>5°) had lower rates of PRRSV outbreaks^49^. However, the area in which our study was conducted only included landscapes with a maximum of ~2.5° slope, which may explain the discrepancy of these results. The model also found that sow farms were at reduced risk when the percent of the neighborhood that was vegetated ranged between 90 and 95%, which is similar to the finding of Arruda *et al*.^49^ that showed that PRRSV outbreaks occurred at lower rates in farms that were surrounding by herbaceous cover and trees relative to cultivated or managed land. However, the increase in risk that we found at extremely high levels of vegetation may be due to the fact that we did not differentiate type of vegetation, and it is possible that neighborhoods with extremely high percent vegetation may be cultivated landscapes.

Limitations of this study include the lack of information on PEDV status for farms other than sow farms and that the study was restricted to one full year. It would be beneficial if movement data was available for several years. Furthermore, we have not yet considered alternate neighborhood sizes. Although our current algorithms based on neighborhood sizes of 10 km performed with considerable accuracy, suggesting that this radius indeed captures important aspects of the risk of PEDV spread, ongoing research will focus on optimizing the size of the neighborhood to maximize accuracy. In addition, our incidence database is comprised of veterinarian-reported outbreaks. While only a small number of veterinarians manage the systems included in this study, it is possible that different veterinarians used different criteria to declare outbreaks. That being said, PEDV is a pathogen with high morbidity and high mortality in swine breeding herds, and the time elapsed between introduction and detection of clinical disease in field settings is between two and five days, after which up to 80% of sows may be clinically affected^18,51,52^. Therefore, we have high confidence of veterinarian reports on the timing of outbreaks.

Lastly, movement data was not available for one of the three swine production systems, which may have reduced our ability to predict outbreaks. However, our model performs well even in the absence of these movements, which suggests our approach is robust and the available data captures important aspects of PEDV dynamics even in the absence of full data. The goal of machine-learning algorithms is not only to produce accurate predictions, but also to provide insights on the predictive nature of the data. In addition, this modeling exercise was performed in the context of the introduction of a new virus to the swine industry, where all farms were probably naïve. Predictions of PEDV in current endemic context, where the initial conditions have shifted, may be more challenging. Lastly, while our general machine learning approach has uncovered novel insights into factors related to the risk of PEDV outbreaks, the specific model built here is tuned to the region in which it was developed and thus cannot be directly extrapolated to a new region. However, our general approach could readily be applied to train models for others regions to account for their unique spatial heterogeneities.

## Conclusion

We applied machine-learning algorithms to a rich dataset of epidemiological and environmental factors with the objective of predicting the occurrence of PEDV outbreaks on sow farms. Predicted probabilities of outbreaks and their contributing factors can be visualized and mapped to contribute to disease surveillance and control efforts. In general, the combination of animal movement dynamics and environmental factors suggests that PEDV outbreaks will be most likely in areas characterized by higher hog density, more pig movements, greater wind speeds and temperatures, less vegetation, and higher altitudes, precipitation and slope. Timely communication of these risks to producers is critical in the prevention of disease spread and targeting of risk mitigation methods (such as air filtration, biosecurity, etc.). Our unique approach also allows us to simultaneously capture disease risks associated with long-distance animal movement and local environmental dynamics. The model presented here forms the foundation for near real-time disease forecasting and will advance disease surveillance and control for endemic swine pathogens in the United States.

## Material and Methods

### Data analysis

#### Descriptive analysis

For every neighborhood *N*, the weekly number of animals and batches moved into *N*, were aggregated according to movement type (Fig. 1-represented by the blue arrows, additional information about data processing is found in Supporting Information S2). A batch is defined as a group of pigs transported from a point A to point B. Descriptive (average, minimum, maximum) weekly summaries of movements by the four movement types (finisher, weaning, breeding, or internal) were calculated for both number of animals and batches. The proportion of movements for each movement type was calculated relative to the total number of movements in 2014. The datasets used for this study are managed by the Morrison Swine Health Monitoring Project (MSHMP). The identity and location of the specific production systems that provided the data has been kept anonymous at the request of the production systems.

### Machine learning

In order to determine the association between PEDV outbreaks in sow farms and possible predictor variables, a set of supervised machine-learning (ML) algorithms: Random Forest (RF)^33,53^, Support Vector Machine (SVM)^54^, and Gradient Boosting Machine (GBM)^55^ were used. Considering the econometric theorem ‘no free lunch’, which states that there is no single best algorithm or “silver bullet” that performs best in all situations^56^, we operated under the assumption that an algorithm that is ideal for one class of problems may perform poorly for other classes of problems^57^. Considering the above, we explored the most popular ML algorithms used for complex problems, RF, SVM and GBM, in order to carefully choose the algorithm with best performance and most suitable for our research question^53^.

#### Data preparation

The outcome modeled through ML was the occurrence (yes) or absence (no) of a new PEDV outbreak within a sow farm during each week in 2014 as a function of environmental, landscape, and movement-related variables (Supplementary Information S1). Overall, 10.5% of sow farms experienced outbreaks at some point during 2014; outbreaks did not occur in 89.5% of sow farms. Given that imbalances between yes/no classes will result in prediction biases in the machine-learning analysis^58^, in that algorithms tend to achieve high overall accuracy by predicting the majority class yet allowing for high error rates for predicting the minority class. Thus, we used a down-sampling strategy via “downSample” function in the R package *Caret*^59^ in which the majority class was randomly down-sampled to match the frequency of the rarest class. Prior to down-sampling, the original data was randomly and uniformly divided into a training (80%) and an independent test set (20%). Data splitting is a common approach used for evaluation and validation of model performance when external data is limited or not available. The training set was used to train the ML algorithms via a k-fold cross-validation process and the quasi-independent test set (20% data not used for model building) was used for validation.

### Algorithm training

The supervised machine learning algorithms RF, SVM and GBM were trained (80% of data) using the complete set of explanatory variables. RF was performed with *randomForest* package^60^, and SVM and GBM were performed with the *caret* package^59^. In the training steps, we adopted a repeated 10-fold cross-validation to better estimate model performance and in order to prevent overfitting and artificial inflation of accuracy due to use of the same data for training and validation steps of the analysis. All algorithms were applied with default parameter setting.

#### Model performance

Model performance was assessed by calculating the total training accuracy, specificity, and sensitivity based on the construction of a confusion matrix. Briefly, the confusion matrix displays the number of observed outbreaks that were correctly (true positive, TP) or incorrectly (false positive, FP) predicted by the model, as well as the number of farms where no outbreak occurred that were correctly (true negative, TN) or incorrectly (false negative, FN) predicted. Accuracy (ACC) was calculated as the overall proportion of observations correctly predicted. Specificity (SPE) was calculated by dividing TN by the sum of TN and FP (reported as a percentage). Sensitivity (SEN) was calculated as TP divided by the sum of TP and FN (reported as a percentage). In addition, the Receiver Operating Characteristic (ROC) curve was graphed for each algorithm and the area under the curve (AUC) was calculated as an additional assessment of model performance.

For each ML algorithm, AUC, accuracy, sensitivity, and specificity were calculated based on the overall average confusion matrix across all folds in the cross-validation. The best algorithm was selected by comparing AUC, accuracy, sensitivity, and specificity for each of the three approaches.

#### Model evaluation on independent data

A crucial step in the evaluation of ML algorithms is to access their prediction performance in independent data. The 20% of the data that was set aside from the original data set was used as a quasi-independent test set of observations. These data were fed into the ML algorithm, allowing the algorithm to predict the outcome for the new data. AUC, accuracy, sensitivity, and specificity were calculated.

#### Variable selection

To rank the importance of each variable in improving the accuracy of model predictions, we used the unscaled version of the function “VarImp” from the *caret* package. Briefly, regardless of the algorithm, the variable’s importance score (i.e., Gini Index) represents how relevant each variable was for predicting PEDV outbreaks, with larger values representing more important predictors. In addition, we calculated the accuracy decrease that quantified the extent to which the accuracy of predictions changed when the variable was permuted relative to the outcome. For the best-fit algorithm (random forest), we examined how often each variable was selected by the algorithm to subdivide the data in the individual decision trees that make up the random forest; each subdivision in the tree is referred to as “node.” A p-value was additionally calculated for each variable to evaluate whether it was used in the random forest more often than if variables were included in decision trees at random. P-values were based on a binomial distribution of the number of nodes split on the variable assuming that variables are randomly drawn^33,34^.

The influence of the most important variables was further analyzed via partial dependence plots, which provide insights on the marginal effect of each predictor on the likelihood of a PEDV outbreak while controlling for the effects of all other variables. The partial dependence of a variable’s effect is best understood by visually examining general patterns in relation to the values of the predictor variable^61^. Because we are modeling binary classification (i.e., presence/absence of PEDV outbreaks), partial dependence values are reported on the “logit” scale and are computed in relation to the probability for the positive class^62^; larger values indicate higher risk of PEDV outbreak.

## Supplementary information

supplementary information

## References

[1] Tomley FM, Shirley MW (2009). Livestock infectious diseases and zoonoses. Philos T R Soc B.

[2] Otake S, Dee S, Corzo C, Oliveira S, Deen J (2010). Long-distance airborne transport of infectious PRRSV and Mycoplasma hyopneumoniae from a swine population infected with multiple viral variants. Vet. Microbiol..

[3] Tatem AJ, Rogers DJ, Hay SI (2006). Global transport networks and infectious disease spread. Adv Parasit.

[4] VanderWaal KL (2016). Network analysis of cattle movements in Uruguay: Quantifying heterogeneity for risk-based disease surveillance and control. Prev Vet Med.

[5] Rossi, G. *et al*. The Potential Role of Direct and Indirect Contacts on Infection Spread in Dairy Farm Networks. *Plos Comput Biol* **13** (2017). 10.1371/journal.pcbi.1005301 PMC5268397 28125610

[6] Smith RP, Cook AJC, Christley RM (2013). Descriptive and social network analysis of pig transport data recorded by quality assured pig farms in the UK. Prev Vet Med.

[7] Motta, P. *et al*. Implications of the cattle trade network in Cameroon for regional disease prevention and control. *Sci Rep-Uk* **7** (2017). 10.1038/srep43932 PMC5339720 28266589

[8] VanderWaal K, Morrison RB, Neuhauser C, Vilalta C, Perez AM (2017). Translating big data to smart data for veterinary epidemiology. Frontiers in Veterinary Science.

[9] Ssematimba, A., Hagenaars, T. J. & de Jong, M. C. M. Modelling the Wind-Borne Spread of Highly Pathogenic Avian Influenza Virus between Farms. *Plos One* **7** (2012). 10.1371/journal.pone.0031114 PMC3279517 22348042

[10] Tago, D., Hammitt, J. K., Thomas, A. & Raboisson, D. The Impact of Farmers’ Strategic Behavior on the Spread of Animal Infectious Diseases. *Plos One* **11** (2016). 10.1371/journal.pone.0157450 PMC4907430 27300368

[11] Brooks-Pollock E, Roberts GO, Keeling MJ (2014). A dynamic model of bovine tuberculosis spread and control in Great Britain. Nature.

[12] VanderWaal K (2017). Optimal surveillance strategies for bovine tuberculosis in a low-prevalence country. Scientific Reports.

[13] Thakur KK, Revie CW, Hurnik D, Poljak Z, Sanchez J (2015). Simulation of between-farm transmission of porcine reproductive and respiratory syndrome virus in Ontario, Canada using the North American Animal Disease Spread Model. Prev Vet Med.

[14] Boender, G. J., van den Hengel, R., van Roermund, H. J. W. & Hagenaars, T. J. The Influence of Between-Farm Distance and Farm Size on the Spread of Classical Swine Fever during the 1997-1998 Epidemic in The Netherlands. *Plos One* **9** (2014). 10.1371/journal.pone.0095278 PMC3991596 24748233

[15] Otake S (2004). Studies on the carriage and transmission of porcine reproductive and respiratory syndrome virus by individual houseflies (Musca domestica). Vet Rec.

[16] Lee K (2017). Unraveling the contact patterns and network structure of pig shipments in the United States and its association with porcine reproductive and respiratory syndrome virus (PRRSV) outbreaks. Prev Vet Med.

[17] Valdes-Donoso P, VanderWaal K, Jarvis LS, Wayne SR, Perez AM (2017). Using Machine Learning to Predict Swine Movements within a Regional Program to Improve Control of Infectious Diseases in the US. Frontiers in Veterinary Science: Veterinary Epidemiology and Economics.

[18] Saif, L. J., Pensaert, M. B., Sestak, K., Yeo, S. & Jung, K. In *Diseases of Swine* (eds Zimmerman, J. J *et al*.) 501–524 (John Wiley & Sons Ltd, 2012).

[19] Goede D, Morrison RB (2016). Production impact and time to stability in sow herds infected with porcine epidemic diarrhea virus (PEDV). Prev Vet Med.

[20] Lee, C. Porcine epidemic diarrhea virus: An emerging and re-emerging epizootic swine virus. *Virol J* **12** (2015). 10.1186/s12985-015-0421-2 PMC4687282 26689811

[21] Kim, Y., Yang, M., Goyal, S. M., Cheeran, M. C. J. & Torremorell, M. Evaluation of biosecurity measures to prevent indirect transmission of porcine epidemic diarrhea virus. *Bmc Vet Res* **13** (2017). 10.1186/s12917-017-1017-4 PMC5382501 28381304

[22] Lowe J (2014). Role of Transportation in Spread of Porcine Epidemic Diarrhea Virus Infection, United States. Emerg Infect Dis.

[23] O’Dea, E. B., Snelson, H. & Bansal, S. Using heterogeneity in the population structure of US swine farms to compare transmission models for porcine epidemic diarrhoea. *Sci Rep-Uk* **6** (2016). 10.1038/srep22248 PMC4780089 26947420

[24] Lee K (2017). Unraveling the contact patterns and network structure of pig shipments in the United States and its association with porcine reproductive and respiratory syndrome virus (PRRSV) outbreaks. Prev Vet Med.

[25] Alonso C (2014). Evidence of infectivity of airborne porcine epidemic diarrhea virus and detection of airborne viral RNA at long distances from infected herds. Vet Res.

[26] Alvarez J, Goede D, Morrison R, Perez A (2016). Spatial and temporal epidemiology of porcine epidemic diarrhea (PED) in the Midwest and Southeast regions of the United States. Prev Vet Med.

[27] Pujols J, Segales J (2014). Survivability of porcine epidemic diarrhea virus (PEDV) in bovine plasma submitted to spray drying processing and held at different time by temperature storage conditions. Veterinary Microbiology.

[28] Dee, S. *et al*. Modeling the transboundary risk of feed ingredients contaminated with porcine epidemic diarrhea virus. *Bmc Vet Res* **12** (2016). 10.1186/s12917-016-0674-z PMC4788872 26968372

[29] Arruda, A. G., Vilalta, C., Perez, A. & Morrison, R. Land altitude, slope, and coverage as risk factors for Porcine Reproductive and Respiratory Syndrome (PRRS) outbreaks in the United States. *Plos One* **12** (2017). 10.1371/journal.pone.0172638 PMC5393554 28414720

[30] Dee, S., Otake, S., Oliveira, S. & Deen, J. Evidence of long distance airborne transport of porcine reproductive and respiratory syndrome virus and Mycoplasma hyopneumoniae. *Vet Res* **40** (2009). 10.1051/vetres/2009022 PMC2701181 19379664

[31] Otake S, Dee S, Corzo C, Oliveira S, Deen J (2010). Long-distance airborne transport of infectious PRRSV and Mycoplasma hyopneumoniae from a swine population infected with multiple viral variants. Veterinary Microbiology.

[32] Sasaki, Y. *et al*. The spatial dynamics of porcine epidemic diarrhea (PED) spread in Miyazaki prefecture, Japan, *Prev Vet Med in press (2017)*. 10.1016/j.prevetmed.2017.05.025 28716208

[33] Breiman L (2001). Random forests. Mach Learn.

[34] Ishwaran H, Kogalur UB, Gorodeski EZ, Minn AJ, Lauer MS (2010). High-Dimensional Variable Selection for Survival Data. J Am Stat Assoc.

[35] Kukielka EA, Martinez-Lopez B, Beltran-Alcrudo D (2017). Modeling the live-pig trade network in Georgia: Implications for disease prevention and control. PLoS One.

[36] Amirpour Haredasht S (2017). Modeling the spatio-temporal dynamics of porcine reproductive & respiratory syndrome cases at farm level using geographical distance and pig trade network matrices. BMC Vet Res.

[37] VanderWaal, K., Perez, A. M., Torremorell, M., Morrison, R. B. & Craft, M. Role of animal movement and indirect contact among farms in transmission of porcine epidemic diarrhea virus. *Epidemics*, in press (2018) 10.1016/j.epidem.2018.04.001 PMC7104984 29673815

[38] Thakur KK, Revie CW, Hurnik D, Poljak Z, Sanchez J (2016). Analysis of swine movement in four Canadian regions: network structure and implications. Transboundary and Emerging Diseases.

[39] Lentz, H. H. K. *et al*. Disease Spread through Animal Movements: A Static and Temporal Network Analysis of Pig Trade in Germany. *Plos One* **11** (2016). 10.1371/journal.pone.0155196 PMC4859575 27152712

[40] Salines M, Andraud M, Rose N (2018). Combining network analysis with epidemiological data to inform risk-based surveillance: Application to hepatitis E virus (HEV) in pigs. Prev Vet Med.

[41] Klinge KL, Vaughn EM, Roof MB, Bautista EM, Murtaugh MP (2009). Age-dependent resistance to Porcine reproductive and respiratory syndrome virus replication in swine. Virology Journal.

[42] Keeling MJ (2005). The implications of network structure for epidemic dynamics. Theor. Popul. Biol..

[43] Kao RR, Green DM, Johnson J, Kiss IZ (2007). Disease dynamics over very different time-scales: foot-and-mouth disease and scrapie on the network of livestock movements in the UK. J R Soc Interface.

[44] Green DM, Kiss IZ, Kao RR (2006). Modelling the initial spread of foot-and-mouth disease through animal movements. Proceedings of the Royal Society of London B.

[45] Beam A (2015). A Porcine Epidemic Diarrhea Virus Outbreak in One Geographic Region of the United States: Descriptive Epidemiology and Investigation of the Possibility of Airborne Virus Spread. PLoS One.

[46] Dee S (2014). An evaluation of contaminated complete feed as a vehicle for porcine epidemic diarrhea virus infection of naive pigs following consumption via natural feeding behavior: proof of concept. BMC Veterinary Research.

[47] Lowe J (2014). Role of transportation in spread of porcine epidemic diarrhea virus infection, United States. Emerging Infectious Diseases.

[48] Pasick J (2014). Investigation into the role of potentially contaminated feed as a source of the first-detected outbreaks of Porcine Epidemic Diarrhea in Canada. Transboundary and Emerging Diseases.

[49] Arruda A, Vilata C, Morrison RB, Perez AMPO (2017). i. r. Land Altitude, Slope, and Coverage as Risk Factors for Porcine Reproductive and Respiratory Syndrome (PRRS) Outbreaks in the United States. PLOS One.

[50] Keeling, M. J. & Rohani, P. *Modelling Infectious Diseases in Human and Animals*. 408 (Princeton University Press, 2008).

[51] Bowman AS, Krogwold RA, Price T, Davis M, Moeller SJ (2015). Investigating the introduction of porcine epidemic diarrhea virus into an Ohio swine operation. BMC Veterinary Research.

[52] CAHFS. Porcine Epidemic Diarrhea Virus (PEDV). (Center for Animal Health and Food Safety, University of Minnesota, St. Paul, MN, 2013).

[53] Machado, G., Mendoza, M. R. & Corbellini, L. G. What variables are important in predicting bovine viral diarrhea virus? A random forest approach. *Vet. Res*. **46** (2015). 10.1186/s13567-015-0219-7 PMC4513962 26208851

[54] Boser, B. E., Guyon, I. M. & Vapnik, V. N. A Training Algorithm for Optimal Margin Classifiers. *Proceedings of the 5th Annual ACM Workshop on Computational Learning Theory* (1992).

[55] Friedman, J. H. Greedy Function Approximation: A Gradient Boosting Machine. *The Annals of Statistics* **29** (1999).

[56] Qiao HJ, Soberon J, Peterson AT (2015). No silver bullets in correlative ecological niche modelling: insights from testing among many potential algorithms for niche estimation. Methods Ecol Evol.

[57] Ho YC, Pepyne DL (2002). Simple explanation of the no-free-lunch theorem and its implications. J Optimiz Theory App.

[58] Guo HX (2017). Learning from class-imbalanced data: Review of methods and applications. Expert Syst Appl.

[59] Kuhn M (2017). caret: Classification and Regression. Training. R: version.

[60] Liaw, A. & Wiener, M. Classification and Regression by randomForest. *R News* **2**(3) (2002).

[61] Friedman JH (2001). Greedy function approximation: A gradient boosting machine. Ann Stat.

[62] Cutler DR (2007). Random forests for classification in ecology. Ecology.

