# Supplementary material for: Identifying outbreaks of Porcine Epidemic Diarrhea virus through animal movements and spatial neighborhoods
Source: Sci Rep. 2019 Jan 24;9:457. doi: 10.1038/s41598-018-36934-8 (PMC6345879; doi:10.1038/s41598-018-36934-8)
Supplement: Supplementary file 1 — supplementary information [file 41598_2018_36934_MOESM1_ESM.docx]

**Identifying outbreaks of Porcine Epidemic Diarrhea virus through animal movements and spatial neighborhoods**

Machado, G^1*^, Vilalta, C.^2^, Recamonde-Mendoza, M.^3^,Corzo, A. C. ^2^, Torremorell, M. ^2^, Perez A.M^2^., VanderWaal, K^2^

**Supporting information S1**. Environmental and climatic factors assessed for association with PEDV outbreaks for tree overlap swine production systems

| **Variable** | **Description (unit)** | **Source** |
| --- | --- | --- |
| Hog density^#^ | Observed livestock densities | Livestock densities**-Website** http://www.fao.org/ag/againfo/resources/en/glw/GLW_dens.html |
| Vegetation^#^ | Values range from 0 (corresponding to 0% vegetation cover) to 100 (corresponding to 100% vegetation cover) | *1 km MODIS-based Maximum Green Vegetation Fraction*-**Website** http://landcover.usgs.gov/green_veg.php |
| Land cover^#^ | The land cover classification from Water to Barren or Sparsely vegetated as described in Broxton et al., 2014 | *0.5 km MODIS-based Global Land Cover Climatology*-**Website** http://landcover.usgs.gov/global_climatology.php |
| Wetlands^#^ | The map was made using the Global Lakes and Wetlands Database (GLWD), which was created using a variety of the best available sources for lakes and wetlands on a global scale | *Wetlands* -**Website** http://www.cec.org/tools-and-resources/map-files/wetlands |
| Global relief (altitude) ^#^ | Altitude (m) | *Global relief based on SRTM DEM and ETOPO*- **Website** https://www.ngdc.noaa.gov/mgg/global/ |
| Global topography (slope) ^#^ | Slope (%) | *Slop map in percent based in the DEMSRE*- **Website** https://www.isric.org/ |
| Average incoming solar radiation^#^ | 8 days average × 365/8 kWh / m2 | Mean potential incoming solar radiation derived in SAGA GIS - **Website** https://www.isric.org/ |
| Average temperature* | Average (°C)- from monthly averages | Average temperature**-Website** http://worldclim.org/version2 |
| Average precipitation* | Mean (mm)- from monthly averages | Mean monthly precipitation**-Website** http://worldclim.org/version2 |
| Topsoil pH^#^ | pH (-log( H+ ions )) | Topsoil pH**-Website** https://www.isric.org/ |
| Major soil^#^ | FAO soil classification system | Spatially domain major soil**-****Website**- http://www.fao.org/soils-portal/soil-survey/soil-classification/en/ |
| Wind speed* | Mean (km/h)-from monthly averages | Global Forecast System (GFS) -**Website** Global Forecast System (GFS) of the USA’s NationalWeather  Service (NWS) (https://www.ncdc.noaa.gov/data-access/model-data/model-datasets/global-forcast-system-gfs). |

^#^Variables that were static (values do not change dynamically in a year) *Variables that were dynamic (calculated monthly mean)

**Supporting information S2**. Workflow for data collection and calculation. For dynamic layers the monthly mean was calculated.


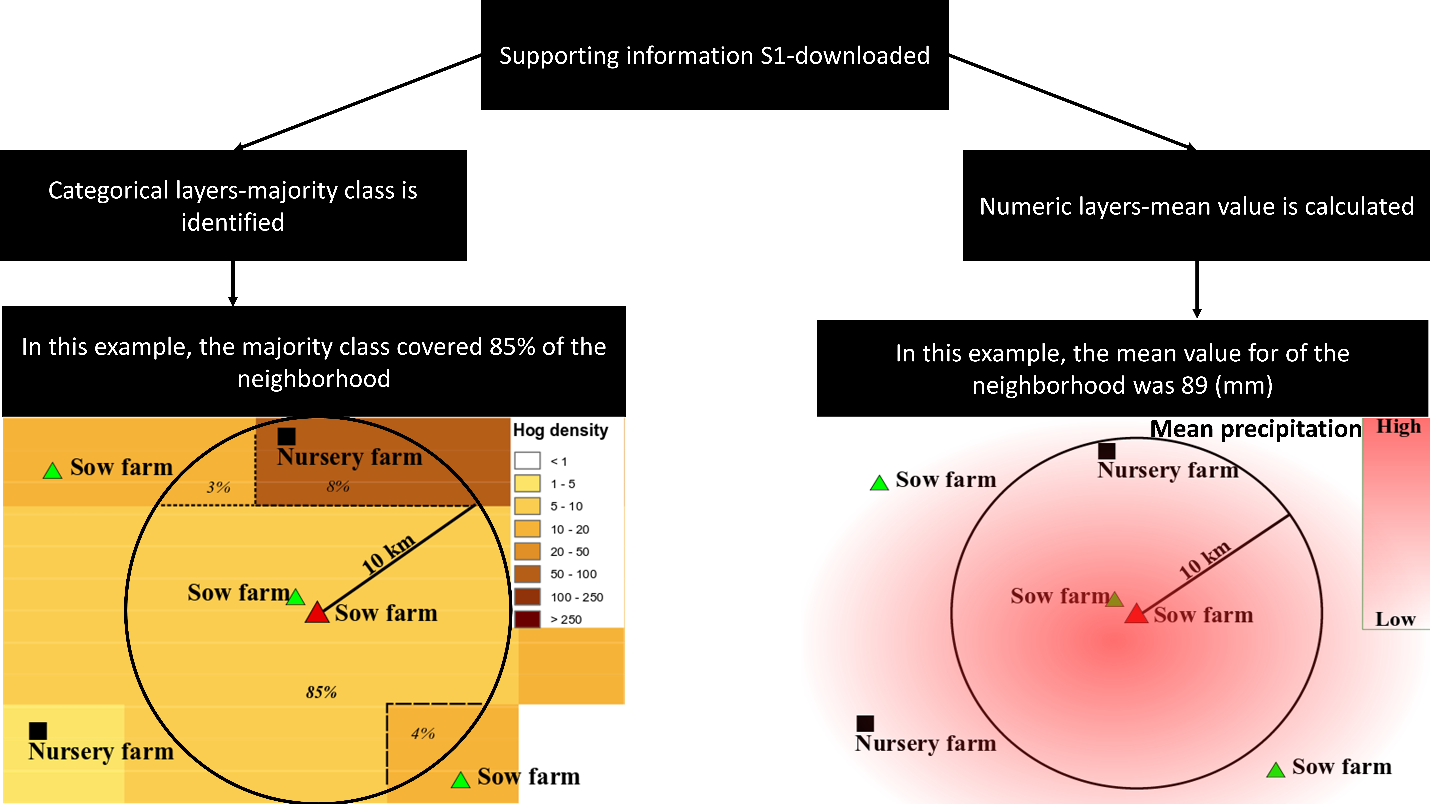


**Supporting information S3**. Descriptive analysis of all variables used for model

| **Variable** | **Values are calculated from the 332 neighborhood** |
| --- | --- |
| Number of pigs moved into buffer zones* | Negative (Median=980-IQR 879)  Positive (Median=926- IQR 800)  All (Median=977- IQR 878) |
| Season of the year# | Fall- Positive (10.57%) Negative (27.37%)  Spring Positive (39.82%) Negative (22.39%)  Summer Positive (31.03%) Negative (24.69%)  Winter Positive (18.56%) Negative (25.53%) |
| Mean win speed (km/h) | Negative (Median=101-IQR=92)  Positive (Median=64.5- IQR=70)  All (Median=94- IQR 92) |
| Mean temperature (F) | Negative (Median=70-IQR=55)  Positive (Median=72- IQR=52)  All (Median=70- IQR=55 |
| Mean vegetation (%) | Negative (Median=86-IQR=83)  Positive (Median=101- IQR=76)  All (Median=90- IQR=84) |
| Mean altitude (m)# | Negative (Median=92-IQR=98)  Positive (Median=90- IQR=65)  All (Median=91- IQR=94) |
| Mean precipitation (mm) | Negative (Median=89-IQR=84)  Positive (Median=108- IQR=97)  All (Median=89- IQR=86) |
| Weaning* | Negative (Median=2-IQR=1)  Positive (Median=2- IQR=1)  All (Median=2- IQR=1) |
| Hog density# | Negative (Median=179-IQR=314)  Positive (Median=175- IQR=471)  All (Median=177- IQR=317) |
| Mean slope# | Negative (Median=0.56-IQR=0.45)  Positive (Median=0.63- IQR=0.36)  All (Median=0.56- IQR=0.45) |
| Finisher* | Negative (Average=0.09-sd=0.43  Positive (Average =0.39- sd=0.84)  All (Average =0.09- sd=0.43) |
| Mean solar | Negative (Median=33.5-IQR=0.49)  Positive (Median=33.4- IQR=0.30)  All (Median=33.53- IQR=0.49) |
| Breeding* | Negative (Average =0.50-sd=1.29)  Positive (Average =0.40- sd=0.49)  All (Average =0.49- sd=1.28) |
| Mean pH# | Negative (Median=4.95-IQR=0.07)  Positive (Median=4.95- IQR=0.11)  All (Median=4.49- IQR=0.07) |
| Land cover# | Class 5- Positive (12.64%) Negative (23.70%)  Class 8-Positive (44.82%) Negative (22.08%)  Class 12- Positive (1.14%) Negative (2.77%)  Class 14- Positive (41.37%) Negative (51.44%) |
| Wetland# | Class 1- Positive (0%) Negative (1.28%)  Class 2-Positive (0%) Negative (0.64%)  Class 10- Positive (2.26%) Negative (8.63%)  Class 11- Positive (97.70%) Negative (89.44%) |
| Sow* | Negative (Average =0.17-sd=0.73)  Positive (Average =0.02- sd=0.21)  All (Average =977- sd=0.72) |
| Major soil# | Class 2- Positive (12.64%) Negative (44.48%)  Class 17-Positive (0%) Negative (1.28%)  Class 27- Positive (58.62%) Negative (47.05%)  Class 28- Positive (28.73%) Negative (7.18%) |

# Static variables; * variables are dynamic, the total numbers of movements per week were considered in the model. Note: average are presented for variables with median near zero.
